# Age and educational track influence adolescent discounting of delayed rewards

**DOI:** 10.3389/fpsyg.2013.00993

**Published:** 2013-12-26

**Authors:** Nikki C. Lee, Renate H. M. de Groot, Annemarie Boschloo, Sanne Dekker, Lydia Krabbendam, Jelle Jolles

**Affiliations:** ^1^Department of Educational Neuroscience and LEARN! Research Institute, Faculty of Psychology and Education, VU University AmsterdamAmsterdam, Netherlands; ^2^Department of Psychiatry and Neuropsychology, School for Mental Health and Neuroscience, Maastricht UniversityMaastricht, Netherlands; ^3^Centre for Learning Sciences and Technologies, Open UniversityHeerlen, Netherlands

**Keywords:** temporal discounting, adolescence, age, development, educational track

## Abstract

This study examined age-related changes in a specific aspect of adolescent decision-making, namely the preference for future versus immediate outcomes. A sample of 622 Dutch adolescents aged 12–17 years completed a temporal discounting task. Participants were asked to choose between a delayed reward of €50 or an immediate reward of lower value. The delay interval was varied in three blocks (1 week, 1 month, 6 months). Results showed that preferences for large delayed rewards over smaller immediate rewards increased with age: late adolescents made more long-term decisions than early adolescents. This change was related to educational track. In the lower educational track, an age-related decrease in discounting was found for all three delay intervals. In the higher educational track this decrease only occurred for the 6 month delay interval. However, across all delay intervals enrolment in a higher level educational track was associated with an increased preference for long-term rewards. These results suggest that late adolescents are less susceptible than early adolescents to the competing presence of an immediate reward when making long-term decisions, a skill which becomes increasingly important as they transition into adulthood.

## INTRODUCTION

Adolescents are often characterized as impulsive and unable to plan ahead or envisage the long-term effects of their behavior. Statistics show that they are more likely than adults to be involved in activities with potentially dangerous consequences such as road traffic accidents, smoking, drug use, or unsafe sexual behaviors (Reyna and Farley, 2006; Steinberg, 2008). Recently, these impulsive behaviors have been examined in adolescent samples using a behavioral paradigm known as temporal discounting (Olson et al., 2007; Olson et al., 2009; Steinberg et al., 2009; Christakou et al., 2011). Temporal discounting tasks measure the decline in the subjective value of a future reward as the time between the decision and the delivery of the reward increases (Ainslie, 1975; Rachlin et al., 1991; Green et al., 1994). Adolescents with a steeper rate of temporal discounting are more driven by immediate gratification than by the long-term consequences of their behavior. For this group the value of the delayed reward therefore decreases strongly as the delay interval increases. This comparison of immediate and delayed rewards makes temporal discounting tasks useful in examining the trade-offs made by adolescents when making reward-related decisions. Adolescence is a period during which many decisions are made that require weighing up both short and long-term costs and benefits, such as deciding between getting a job or going to university, or between spending a weekend studying for an important exam or going out with friends. Therefore, it is important to understand how the ability to choose between immediate and delayed rewards develops during adolescence, as these skills may be vital to ensure a successful transition to adulthood.

Within a temporal discounting task, a participant often completes multiple trials with consistent delayed rewards but varying delay intervals (e.g., “Would you prefer €10 today or €15 tomorrow, next week, next month, next year?”). The data resulting from these repeated trials gives an estimation of the rate at which the subjective value of the delayed reward decreases in value over time, i.e., is discounted. This is known as the *discount rate* (Myerson and Green, 1995). The subjective value of the delayed reward for each time point is known as the *indifference point*, and is equal to the amount at which the participant finds the larger future and smaller current reward of equal value (Myerson et al., 2001). In adolescents, higher rates of discounting have been found in specific groups such as smokers compared to non-smokers (Reynolds and Fields, 2012), and heavy drinkers compared to light drinkers (Field et al., 2007), as well being a characteristic of certain developmental disorders such as attention deficit hyperactivity disorder (Barkley, 1997) and conduct disorder (Anokhin et al., 2011).

A few studies have examined developmental changes in discounting behavior during adolescence. Initial studies often compared a single group of adolescents of diverse ages to groups of young or older adults. For example, Green et al. (1999) collected data from a group of young adolescents and found them to discount the value of delayed rewards more steeply then a group of college students and a group of older adults. A subsequent study by Scheres et al. (2006) examined changes during the adolescent period in more detail. They compared groups of primary and secondary school children and found that the younger group discounted significantly more strongly than the older group. Other studies using a similar age range have demonstrated comparable results (e.g., Olson et al., 2007). However, these studies used broad age ranges, and were therefore unable to examine if these changes occur across the entire adolescent age range, or are limited to a specific period in adolescent development. A recent study by Steinberg et al. (2009) examined these changes in more detail. They found that young adolescents, aged 13 and younger, discounted significantly more steeply than adolescents aged 16 and older, with 14- and 15-year-olds falling somewhere in between. No significant age differences were found in the older age groups, which ranged from 17 to 30 years of age.

Individual differences that may influence discounting behavior, such as sex and educational attainment, have previously been examined in adult samples. Higher income and education have been associated with lower discount rates (Harrison et al., 2002; Jaroni et al., 2004; De Wit et al., 2007). Discount rates have been shown to have a negative association with grade point average in university students (Silva and Gross, 2004; Kirby et al., 2005). Previous studies of sex differences have reported varying findings, making it difficult to draw overall conclusions on sex differences in discount rates (Kirby and Marakovic, 1996; Harrison et al., 2002; Silverman, 2003; Reynolds et al., 2006; Reimers et al., 2009; Steinberg et al., 2009). In adolescents, the effects of individual differences have not been studied extensively.

When taken together, the results of the aforementioned studies suggest that discounting behavior continues to develop during adolescence and may be influenced by individual differences. However, these studies have analyzed differences in discounting behavior by comparing only one general discounting metric such as the discount rate. Such metrics summarize intertemporal preferences measured across several delay intervals into one quantitative variable, and are therefore unable to indicate at which subset of the delay intervals the biggest changes occur. However, a recent study in adults and adolescents showed that differences between them in discounting behavior became more apparent as delay intervals were increased (Christakou et al., 2011). This suggests that examination of indifference points, which reflect intertemporal preferences at specific delay intervals, will lead to a better understanding of exactly how adolescent development affects decision-making at varying intervals between the decision and its consequences. For example, adolescent A may have a higher discount rate than adolescent B, suggesting that he is always more drawn to immediate rewards. However, this higher discount rate could be the result of a higher preference for immediate rewards for long delay intervals (e.g., 6 months) not short delay intervals (e.g., a week). In fact, adolescent A’s indifference points for short delay intervals could be identical to those of adolescent B, with only his indifference points for long delay intervals causing the differences between them in discount rate. However, this can only be established through examination of indifference points and not through comparison of discount rates. By examining indifference points in this study, we hope to draw conclusions about in which delay intervals the temporal aspects of the decision are most influential during adolescence.

The aims of the current study are threefold. First, we aim to further examine the previously mentioned findings of Steinberg et al. (2009). We will use a large sample comprising three age groups (12–13, 14–15, and 16–17 years) so that close evaluation of developmental trajectories is possible. Secondly, we aim to extend previous findings by examining differences in discounting behavior at three specific time points to see if behavior differs between the groups when the time between the immediate and delayed reward is a week, month, or 6 months. Developmental differences in indifference points have not been examined in previous studies, but will elucidate the causes of developmental changes in the previously examined discount rates. And finally, we will analyze the role of individual differences between adolescents, namely sex and educational track, on discounting behavior.

To this end, a large cross-sectional sample (*N* = 622) of Dutch secondary school pupils, enrolled in the two highest educational tracks, completed a temporal discounting task. We hypothesize that discounting rates will decrease with age, i.e., that participants will become decreasingly oriented toward immediate rewards. We expect that this change will differ between the different delay intervals. More specifically, we expect changes in discounting behavior to be positively associated with delay intervals: the longer the interval the larger the decrease in discounting with age. Furthermore, we anticipate that individual differences, such as level of education and sex, will influence this development. In line with previous research we expect that pupils enrolled in a lower educational track will report steeper discounting than those in a higher educational track. As previous research regarding sex differences in discounting behavior has been mixed, we included sex in our model without making a specific hypothesis about the expected direction of sex effects.

## MATERIALS AND METHODS

### PARTICIPANTS

The initial sample included 691 adolescents between the ages of 12–17 years, recruited within a larger research project examining adolescent cognitive development (e.g., Boschloo et al., 2012; Dekker et al., 2013). Data from this project concerning the influence of temporal discounting on academic motivation and achievement have been published elsewhere (Lee et al., 2012). To be included in the sample for the present study, participants had to be typically developing with no prior history of neurological, psychological, and/or psychiatric conditions. Application of these criteria led to exclusion of 37 participants, yielding a sample of 654 participants for the current analyses. Participants were divided into three age groups: early adolescents aged 12–13, mid-adolescents aged 14–15, and late adolescents aged 16–17. Participant characteristics following exclusion are presented in **Table 1**. All participants were enrolled in either senior general secondary education (*hoger algemeen vormend onderwijs* or “*havo*”) or pre-university education (*voorbereidend wetenschappelijk onderwijs* or “*vwo*”). Pupils from the lowest educational track were not recruited for this study, due to the high rate of learning disorders and behavioral problems among this group, which could have confounded the results. The selected tracks constitute the two highest educational tracks within the Dutch system and approximately 40% of pupils are enrolled in one of these tracks. Students are placed in tracks based on their performance on national standardized tests at the end of primary school. Successful completion of senior general education enables the student to enrol at a university of applied science, which offer vocational training in subjects such as nursing, performing arts, and social work. Pre-university education offers entry to higher level programes at research universities, such as medicine and law. Courses at these institutions are more research-oriented than those at universities of applied science, where courses are more practice-oriented.

**Table 1:** Participant characteristics (following exclusion).

	**Age**	**Male: female ratio**	**Level of education**		***N***
	***M* (SD)**		**Senior general education (*N*)****	**Pre-university education (*N*)****	
Age 12–13	12.62 (0.49)	104:117	105	116	221
Age 14–15	14.36 (0.48)	104:148	123	129	252
Age 16–17	16.48 (0.50)	71:121	82	110	192
Total	14.39 (1.60)	279:386	310	355	665

The VU University Amsterdam institutional ethical review board approved all procedures. Written informed consent was obtained from both participants and their parents prior to participation in the study.

### PROCEDURE

Researchers visited selected schools and gave a short presentation to the pupils about the research project. All pupils received an information package to take home, containing information about the project, a consent form and a questionnaire to be filled in by one of the child’s parents or caretakers. Pupils returned the questionnaire and consent form a week later if they wished to participate. During this second session, pupils completed questionnaires and tasks in class under supervision of two trained psychologists and a classroom teacher. Completion of tasks and questionnaires took approximately 40 min, of which 5 min were spent on the temporal discounting task. All pupils who returned the information package were included in the testing procedure. Participants who did not meet the inclusion criteria were removed from the analyses at a later date.

### MEASURES

#### Demographics

By means of a questionnaire filled in by the child’s parents or caretakers, information was gathered about the child’s medical history and educational background. This was used to identify participants who did not meet the inclusion criteria.

#### Temporal discounting

A written version of a temporal discounting task, based on the procedure used by Rachlin et al. (1991) was used to measure temporal discounting behavior. The current task required participants to choose between a fixed delayed reward of €50 and a variable immediate reward of €5, €10, €15, €20, €25, €30, €35, €40, or €45. Three interval lengths were used for the time between the immediate and delayed rewards: 1 week, 1 month, and 6 months. Choices were presented in three blocks: one per delay interval. Within each block choices were presented as separate items, and in ascending order of each of the values of the immediate reward. This resulted in 27 trials per participant. The week delay interval was presented first, followed by the month and 6 month intervals. Responses were used to determine each participant’s indifference point for the three delay periods, defined as the item where participants switched from selecting the delayed reward to selecting the immediate reward. Lower indifference points indicate less willingness to wait for the delayed reward.

The value of the delayed rewards were selected to closely resemble amounts the adolescents could realistically receive, as previous research has shown that the rate of temporal discounting is influenced by reward magnitude (Green et al., 1997). All rewards were hypothetical, as comparisons of tasks using real and hypothetical rewards have shown no difference in the results found (Johnson and Bickel, 2002; Madden et al., 2003, 2004). The delay intervals used in the task were chosen to reflect delay intervals adolescents were likely to have previously experienced when making decisions in real life.

### ANALYSES

The area under the curve (AUC) method was used to calculate a measure of overall discounting behavior and enable comparison with previous studies. This approach is frequently used within experimental research paradigms (e.g., Dixon et al., 2003; Scheres et al., 2006; Olson et al., 2007) as it avoids the difficulties associated with methods based on theoretical discounting functions (Myerson et al., 2001). Participants’ indifference points were normalized, i.e., the delay was recalculated as a proportion of the maximum delay of 6 months and the value of the indifference point was recalculated as a proportion of the maximum reward of €50. Using these normalized values the three indifference points were plotted against (time to) delay. Vertical lines were drawn from each data point to the *x*-axis, thereby creating three trapezoids. The area under the resulting curve was calculated by summing the areas of these three trapezoids [see Myerson et al. (2001) for more information on the procedure]. Due to normalization of the data points the AUC values range between 0.0 and 1.0, with smaller values indicating steeper discounting (i.e., less willingness to wait as time increases).

All effects are reported as significant at *p* < 0.05. To enable comparison of our data with previous research and to measure general discounting behavior, our first analysis comprised a three-way analysis of variance (ANOVA) using age, sex, and educational track as independent variables and the total area under the discounting curve as a dependent variable. Subsequently, in our second analysis, changes in discounting behavior over time were examined using a repeated measures ANOVA, with individuals’ three indifference points (week, month, 6 months) as the within-subjects factor and age, sex, and educational track as between-subjects factors. Mauchley’s test indicated that the assumption of sphericity had been violated, χ^2^(2) = 86.186, *p* < 0.001. Therefore, degrees of freedom were corrected using Greenhouse–Geisser estimates of sphericity (ɛ = 0.886). To further examine the effects of the

individual difference variables at each delay interval, three separate three-way analyses of variance were performed using each of the three individual indifference points as dependent variables and age, sex and educational track as independent variables. In all analyses, significant main effects were further examined using *post hoc* Bonferroni-adjusted pairwise comparisons where appropriate. Significant interaction effects were investigated using *post hoc* simple effects analyses.

## RESULTS

Participants who produced inconsistent discounting data (*N* = 32) were excluded from further analysis, in line with methods used in previous studies (Reynolds et al., 2006; Olson et al., 2007)^1^. Inconsistent discounting was defined as an increase in subjective value as time increased. Analysis showed that consistent and inconsistent discounters did not differ with regard to age, sex, or level of education.

### ANALYSIS 1: AREA UNDER THE CURVE

A three-way ANOVA showed a significant main effect of age on the area under the discounting curve [*F*(2, 622) = 7.667, *p* < 0.001, partial η^2^ = 0.02], with *post hoc* tests indicating that participants in the youngest age group discounted rewards more strongly than those in the oldest age group (*p* < 0.001). A significant main effect of educational track was also found [*F*(1,622) = 31.53, *p* < 0.001, partial η^2^ = 0.05], due to stronger discounting by participants in the lower track, compared to the higher track (see **Table 2**). There was no significant difference in discounting behavior between boys and girls (*p* = 0. 86) and there were no significant interactions.

**Table 2:** AUC and indifference points as a function of age and level of education.

	**Total sample**	**12–13 years**		**14–15 years**		**16–17 years**	
		**Senior general education**	**Pre-university education**	**Senior general education**	**Pre-university education**	**Senior general education**	**Pre-university education**
	***M* (SD)**	***M* (SD)**	***M* (SD)**	***M* (SD)**	***M* (SD)**	***M* (SD)**	***M* (SD)**
AUC	0.59 (0.26)	0.45 (0.26)	0.62 (0.24)	0.56 (0.25)	0.62 (0.23)	0.58 (0.26)	0.64 (0.24)
Indifference point week	€43.15 (8.42)	€39.05 (12.49)	€44.32 (5.85)	€42.95 (8.42)	€43.86 (7.87)	€43.86 (7.41)	€44.66 (5.78)
Indifference point month	€34.37 (14.16)	€26.53 (16.46)	€36.32 (13.28)	€33.42 (14.32)	€36.54 (12.26)	€34.53 (14.03)	€38.13 (11.90)
Indifference point six months	€20.34 (16.76)	€14.23 (14.57)	€21.48 (16.79)	€17.97 (16.21)	€21.52 (16.14)	€18.61 (16.98)	€27.47 (17.27)

### ANALYSIS 2: INDIVIDUAL INDIFFERENCE POINTS

Subsequently a repeated measures ANOVA was performed. Tests of within subject factors showed a significant main effect of delay interval [Greenhouse–Geisser *F*(1.77,1102.71) = 829.03, *p* < 0.001, partial η^2^ = 0.57] with participants discounting more strongly as the delay interval increased. A significant interaction was found between delay interval and educational track [Greenhouse–Geisser *F*(1.77,1102.71) = 7.47, *p* = 0.001, partial η^2^ = 0.01], showing that pupils in the two tracks differed in their changes in discounting behavior over the three delay intervals. The interaction between delay interval, age, and educational track showed a trend toward significance [Greenhouse–Geisser *F*(1.77,1098.32) = 2.34, *p* = 0.06]. No other significant interactions were found.

Tests of between-subject effects showed a significant main effect of age [*F*(2,622) = 8.24, *p* < 0.001, partial η^2^ = 0.03] and educational track [*F*(1,622) = 31.83, *p* < 0.001, partial η^2^ = 0.05], indicating that on average age groups and school types differed in their discounting behavior across time points. The main effect of sex was not significant (*p* = 0.95). The interaction between age and education track showed a trend again toward significance [*F*(2,622) = 2.73, *p* = 0.07]. Other interactions were not significant.

#### Post hoc analyses week and month delay intervals

**FIGURE 1:**
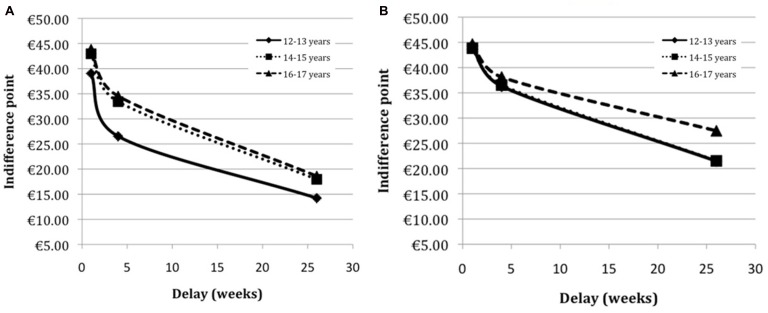
**Age differences in temporal discounting among senior general education (havo) pupils (A) and pre-university (vwo) pupils (B)**.

Findings from follow-up three-way analyses of variance for the week and month delay intervals found significant main effects of age [week: *F*(2,622) = 4.90, *p* = 0.008, partial η^2^ = 0.02; month: *F*(2,622) = 6.79, *p* = 0.001, partial η^2^ = 0.02] and educational track [week: *F*(1,622) = 12.71, *p* < 0.001, partial η^2^ = 0.02; month: *F*(1,622) = 25.15, *p* < 0.001, partial η^2^ = 0.04]. The main effect of sex was not significant (week: *p* = 0.68, month: *p* = 0.93). Additionally, there was a significant age group and educational track interaction [week: *F*(2,622) = 4.61, *p* = 0.01, partial η^2^ = 0.02; month: *F*(2,622) = 3.61, *p* = 0.03, partial η^2^ = 0.01]. *Post hoc* simple effects analyses were used to further examine this interaction. As **Figure 1** shows, the pattern of age differences was not the same across the two educational tracks. For both the week and month delay intervals significant age effects were only present within the lower educational track (week: *p* < 0.001, month: *p* < 0.001). In both conditions the early adolescents discounted more than mid adolescents (week: *p* = 0.01, month: *p* = 0.003) and late adolescents (week: *p* = 0.004, month: *p* = 0.001). No age differences were found within the higher educational track for the week (*p* = 0.86) or month (*p* = 0.68) delay intervals.

#### Post hoc analyses 6 month delay interval

In contrast, for the 6 month delay interval only the main effects of age [*F*(2,622) = 5.58, *p* = 0.004, partial η^2^ = 0.02] and educational track [*F*(1,622) = 24.12, *p* < 0.001, partial η^2^ = 0.04] were significant. The main effect of sex was not significant (*p* = 0.80) and there were no significant interactions. *Post hoc* tests showed that for the 6 month delay interval, the eldest participants discounted rewards less than the youngest participants (*p* = 0.002) and showed a trend toward discounting less than the mid-adolescent group (*p* = 0.06). Those in the higher educational track discounted less across all delay intervals than those in the lower track.

## DISCUSSION

In this study we examined changes in temporal discounting during adolescence related to age, sex, and educational track. Analyses using a single discounting metric, the AUC, showed that discounting decreased with age and educational track: participants in the early adolescent group and those in the lower educational track were more drawn to immediate rewards than those in the late adolescent group and the higher educational track. Thus for younger participants and those enrolled in a lower educational track, the delayed reward lost its subjective value more quickly than for older participants and those enrolled in the higher educational track. The examination of individual indifference points showed that for the week and month delay intervals, pupils enrolled in pre-university education, the higher educational track examined in this study, consistently discounted the value of delayed rewards less than those in the lower track studied, senior general secondary education. However, only those in the lower track showed a decrease with age, indicating that their discounting behavior became increasingly similar to that of the pupils in the higher level. In the case of the 6 month delay interval, age-related decreases were again found, but these did not differ between the two educational levels. No differences were observed between the sexes.

Our additional analyses of individual indifference points clearly indicated the added value of examining indifference points in combination with area under the curve measures. The analyses using area under the curve measures showed that discounting behavior decreased with age, but examination of indifference points showed that this development differs per delay interval. This was in line with our hypotheses, and similar to results previously found in a comparison between adults and adolescents (Christakou et al., 2011). Our finding of developmental changes in discounting behavior concurs with the results of other studies, which have previously reported differences in discounting behavior across the lifespan (Green et al., 1994, 1999; Scheres et al., 2006). In a previous study in adolescents, Steinberg et al. (2009) also found significant differences between young adolescents aged 13 and younger and older adolescents aged 16 and older. Both their data and our findings suggest that the period between 13 and 16 years of age may be important for the development of discounting behavior, and therefore the preference for delayed versus immediate rewards.

These observed age-related changes in discounting behavior could be a result of older adolescents having experienced more temporal delays. This may lead to a more advanced subjective perception of a delay interval: a month may seem shorter to an older adolescent than to a younger individual who has less personal experience with opting for a delayed reward. Previous research in animals has shown that temporal discounting is influenced by familiarity with making decisions between delay intervals (Logue et al., 1984). However, experience of receiving delayed rewards does not explain why changes in discounting behavior occur specifically during the adolescent period. This may be due to the increased salience during adolescence of immediate rewards. This reward sensitivity is known to peak during adolescence compared to childhood and adulthood (Casey et al., 2008; Somerville et al., 2011). Combined with the relative immaturity of self-control processes (Luna et al., 2004), due to continued structural and functional development of the adolescent brain (Giedd, 2004; Gogtay et al., 2004), this results in a vulnerability toward decision-making behavior that is motivated by a desire for immediate gratification.

Recent studies have investigated the relationship between brain development and discounting behavior. Christakou et al. (2011) showed that the previously reported age-related decreases in impulsive choices during adolescence were associated with changes in activation in the limbic corticostriatal network in the brain, including the ventromedial prefrontal cortex. Other work has shown that less impulsive temporal discounting behavior during adolescence was associated with more mature patterns of white matter organization in the lateral prefrontal and temporal and parietal areas of the brain previously implicated in discounting behavior (Olson et al., 2009). Interestingly, some of the reported associations were age-dependent, meaning that they likely reflect developmental processes, while others, particularly in the left temporal and right frontal regions, were age-independent. The authors speculate that these age-independent associations may reflect individual differences in discounting behavior between adolescents. Our results suggest that these individual differences may be associated with the educational track the adolescent is enrolled in.

The discovery that pupils in the lower educational track showed steeper discounting, could be related to studies showing that higher intelligence is associated with lower levels of discounting (Shamosh and Gray, 2008; Shamosh et al., 2008). This suggests that a pupil’s IQ may be one factor influencing the differences in discount rates across educational tracks. However, in the Dutch educational system, pupils are placed in a particular educational track based on their academic achievement and not based on IQ measures. This means that underachieving students may be placed in a lower track than their IQ warrants. This was confirmed by previous research using Dutch pupils, which showed that although IQ is strongly related to educational track, there is also a group of pupils who have similar IQ scores but differ in the educational track they are enrolled in (van den Bos et al., 2012). Thus IQ may not be the sole cause of the lower discount rates in the higher level of education group. Previous studies have shown that lower levels of delay discounting are also associated with higher levels of academic achievement and motivation (Silva and Gross, 2004; Kirby et al., 2005; Lee et al., 2012). Students with higher levels of academic motivation may be more oriented toward their future, leading them to discount its value less, as well as strive to perform better academically. However, the role of IQ in this relationship is unclear, and further research needs to examine the relationship between temporal discounting and academic achievement controlled for IQ.

Through our additional analyses of individual indifference points we were able to show that the developmental changes evidenced by area under the curve measures did not occur for all indifference points. Though early and late adolescents may have similar indifference points when choosing between a delayed amount available next week or a smaller amount available immediately, if the delay interval is increased to 6 months their decisions will differ. This could have behavioral implications that are, relevant in educational settings. For example, passing end of year exams often relies not only on studying the week before the test, but on continuous work throughout the school year to ensure that the pupil is familiar with all concepts that are covered. Now imagine both individuals are told that they need to study for an important test that they must pass, but are also invited to go to a friend’s birthday party. When the amount of time between the test and the party is 1 week, both a 12-year-old and a 16-year-old individual may make a similar decision and decide to study. But if the delay interval is increased to a month their decisions may differ due to an age-related improvement in their decision-making abilities: the 16-year-old may choose to study and the 12-year-old may go to the party. Younger pupils may therefore benefit from assistance in making decisions regarding long-term planning.

A further advantage of the examination of individual difference points is demonstrated by our finding of an interaction between age and level of education. The conventional area under the curve analysis we initially performed showed that discounting behavior improved with age and level of education. However, the analysis of individual indifference points showed that the improvement with age for the two shorter delay intervals only occurred among pupils within the lower level of education. Thus young pupils in the lower educational track are more apt to make impulsive decisions than young pupils in the higher track, who have a greater tolerance for short delays.

In contrast to the AUC measure, our analysis of indifference points also showed continued development of discounting abilities among pupils in the lower level of education compared to the higher level of education. With increasing age, the participants in the lower level of education become more similar in their discounting behavior to participants in the higher level. This finding is in line with intervention studies which suggest that self-control is malleable, and can be improved, for example through training (Diamond and Lee, 2011). As our findings are based on a cross-sectional sample, it is unclear if these developmental changes would result in changes in the rank-order of discounting abilities with age. Alternatively, they could reflect an age-related decrease in variance in discounting behavior, without affecting the rank-order of this ability. Previous studies have shown mixed support for the idea that self-control is a biologically based trait and that despite changes in self-control over time, a child with relatively weak self-control will grow up to be an adult who finds it more difficult than others to delay gratification. While some studies have found evidence of heritability of discounting during adolescence (Anokhin et al., 2011; Mitchell, 2011), previous studies of the broader concept of self-control abilities have suggested that there is only a degree of rank-order stability of these abilities between individuals (Moffitt et al., 2011). More research, using longitudinal samples, is needed to examine the rank-order stability of discounting behavior in more detail.

A number of considerations in interpreting the current results should be mentioned. Though we examined two levels of education, all participants in our study were enrolled in relatively high levels of education, which would enable to them to enter higher education at college or university level. This means that generalization of results to other populations should occur carefully. However, the finding that the greatest development in discounting abilities across delay intervals occurred among pupils in the lower level of education used in our study indicates that larger developmental effects may be found if a less highly educated sample were examined. Furthermore, we cannot exclude the possibility that the order of presentation of the items in the discounting task influenced the results. Previous research (e.g., Robles and Vargas, 2008; Robles et al., 2009), comparing both ascending and descending items, found greater discounting when participants answered questions in ascending order compared to descending order. As the items in our task were only presented in ascending order, our results may have overestimated participants’ discount rates. However, as we do not expect the effect of order of presentation to differ between the groups, this should not have affected the observed group differences.

In conclusion, our results confirm that there are age-related decreases in discounting of delayed relative to immediate rewards, which are influenced by individual differences between adolescents, such as level of education. Additionally, we have shown that the analysis of divergence in individual indifference points can provide additional information when used alongside traditional AUC and discount rate approaches.

## Footnotes

1 The results of the analyses did not differ when inconsistent discounters were included. However, for sake of clarity only the results of the analyses without these participants are reported in this paper.
